# When and why do children develop hyperglycemia during acute lymphoblastic leukemia therapy?

**DOI:** 10.1590/1806-9282.20240953

**Published:** 2025-10-17

**Authors:** Lívia Cristina Oliveira e Silva, Adriana Aparecida Siviero-Miachon, Ana Virgínia Lopes Sousa, Angela Maria Spinola-Castro

**Affiliations:** 1 Universidade Federal de São Paulo, Escola Paulista de Medicina, Division of Pediatric Endocrinology – São Paulo (SP), Brazil.; 2 Hospital of the Support Group for Adolescent and Children with Cancer – São Paulo (SP), Brazil.

**Keywords:** Acute lymphoblastic leukemia, Hyperglycemia, Glucocorticoids, Asparaginase, Oncology

## Abstract

**OBJECTIVE:**

To identify risk factors for hyperglycemia and evaluate the impact on outcomes among pediatric patients with acute lymphoblastic leukemia undergoing chemotherapy.

**METHODS:**

We conducted a retrospective cohort study involving 188 pediatric patients treated for acute lymphoblastic leukemia at a Brazilian cancer referral center between 2004 and 2017. Hyperglycemia was assessed during the induction and consolidation phases of chemotherapy. Associations with patient and disease characteristics were examined through multivariate analysis. Outcomes analyzed included severe infections, relapse, and mortality.

**RESULTS:**

Hyperglycemia occurred in 43.6% of patients, predominantly during the induction phase. Pubertal status at diagnosis, younger age, and high relapse risk classification were independently associated with increased hyperglycemia risk. Puberty conferred a nearly 8-fold increased risk. However, hyperglycemia was not associated with increased risk of infections, relapse, or mortality. Higher mortality was observed among patients with overweight/obesity and those with pancreatitis or recurrent infections.

**CONCLUSION:**

Hyperglycemia was a frequent early adverse event in pediatric acute lymphoblastic leukemia treatment, particularly among pubertal patients and those at high relapse risk. Despite its high incidence, hyperglycemia was not associated with worse clinical outcomes in this cohort. These findings underscore the importance of early identification and monitoring, particularly during high-risk treatment phases.

## INTRODUCTION

Acute lymphoblastic leukemia (ALL) stands as the most prevalent malignancy in the pediatric population, accounting for approximately 25% of diagnoses in individuals up to 15 years of age. The advent of enhanced therapeutic modalities has significantly bolstered survival rates; however, survivors may grapple with a spectrum of adverse effects, including endocrine and metabolic disturbances^1^.

Hyperglycemia is a prevalent early adverse effect of ALL therapy, impacting between 3.8 and 58% of pediatric patients^2–6^. The incidence of hyperglycemia depends on a combination of predisposing patient factors, disease characteristics, and the administration of glucocorticoids and asparaginase (ASP)^2,3^.

The treatment of ALL is governed by various international protocols, with the induction phase being particularly critical for hyperglycemia because of the concurrent administration of glucocorticoids and ASP^2,3^. Diminished insulin secretion or action can disrupt glucose metabolism, while acute pancreatitis, a notable adverse effect of ASP, may further exacerbate hyperglycemia^6,7^.

Moreover, hyperglycemia during ALL treatment may be linked to complications such as infections, disease recurrence, and mortality, serving as a poor prognostic indicator in critically ill patients^8–10^.

While numerous studies have identified predisposing factors for hyperglycemia, we aimed to reassess these associations in a Brazilian pediatric cohort, where treatment protocols and population characteristics may influence outcomes. Accordingly, this study aims to identify the predisposing factors for hyperglycemia during ALL chemotherapy and to determine at-risk patients for timely intervention and follow-up.

## METHODS

### Study population

The study included all patients treated for ALL at the Hospital of the Support Group for Adolescents and Children with Cancer (GRAACC) in São Paulo, Brazil, between 2004 and 2017.

### Procedures

Retrospective data were meticulously gathered from an anonymized database derived from electronic medical records between June 2020 and March 2021. The study received approval from the Ethics Committee of GRAACC (023/2019) and the Federal University of São Paulo–UNIFESP/EPM (4.055.231).

The following variables were examined:

- Chronological age at diagnosis (years);
- Gender: female or male;
- Ethnicity: Caucasian and non-Caucasian;
- Puberty stage at diagnosis: prepubertal and pubertal, according to Tanner stage and hormonal evaluation^11,12^;
- Nutritional status: underweight, healthy, overweight, or obese, according to the World Health Organization body mass index z-scores reference^13^;
- Family history of diabetes mellitus (DM): yes or no;
- Cellular lineage of ALL: B, T-cell, or mixed;
- Presence of Philadelphia chromosome (Ph+);
- Relapse risk-stratified into low, intermediate, or high risk for relapse, according to biologic and clinical prognostic factors for the 2009 Brazilian Group for the Treatment of Leukemia in Childhood (GBTLI-2009)^14^ and the Berlin-Frankfurt-Munich (BFM) treatment protocols^15,16^, which include white blood cell count, age, immunophenotype, treatment response, and unfavorable genetic aberrations;
- Incidence of severe infection episodes, defined as positive culture and requirement for intravenous (IV) antibiotic therapy;
- Elevated pancreatic enzymes: any increment of amylase or lipase;
- Hyperglycemia, as per International Society for Pediatric and Adolescent Diabetes criteria, assessed through random serum glucose ≥11.1 mmol/L or two fasting serum glucose measurements ≥7.0 mmol/L^17^.

Hyperglycemia was assessed during the induction and consolidation phases, which are considered high-risk periods for hyperglycemia due to the concurrent use of glucocorticoids and ASP, and during which fasting capillary blood glucose tests were conducted daily. Serum blood glucose levels were then measured weekly or more frequently, depending on the results of the capillary tests. For the hyperglycemic cohort, additional analyses included insulin requirements and adverse prognosis events, such as severe infections, ALL relapse, and mortality due to ALL or secondary complications.

Two distinct treatment protocols were employed at different time points: GBTLI-2009^14^ and the BFM protocol^15,16^. The cumulative doses of ASP (*Escherichia coli* native or pegylated, PEG) and glucocorticoids (prednisone, PRED, or dexamethasone, DEX) were recorded in international units per square meter (IU/m^2^) or milligrams per square meter (mg/m^2^), respectively.

### Statistical analyses

Data were initially subjected to descriptive analysis. Categorical variables were presented as absolute and relative frequencies, while numerical variables were summarized using mean and standard deviation (SD).

Comparative analyses were conducted between euglycemic and hyperglycemic groups and across different therapeutic protocols using the chi-square or Fisher's exact test for categorical variables and the Student's t-test or Mann-Whitney test for numerical variables.

Hyperglycemia was analyzed as a binary outcome using univariate and multivariate logistic regression models. The adequacy of the logistic model was assessed using the Hosmer and Lemeshow test (p>0.05).

Risk factors for infection were also analyzed, and the effect of individual characteristics on infection incidence was assessed using univariate and multivariate ordered logit regression, stratified by the number of infection episodes during ALL treatment. The multivariate model also modeled hyperglycemia and pancreatitis as time-dependent covariates.

Fine and Gray competing risk models were utilized to evaluate ALL relapse, with univariate followed by multivariate models. The independent variables mirrored those analyzed for infection episodes and elevated pancreatic enzymes. Univariate Cox models were adjusted for all predictor variables, followed by a multivariate model for mortality.

All statistical tests were established at a significance level of 5%. Analyses were conducted using SPSS 20.0 and STATA 12 statistical software.

## RESULTS

### Study population

One hundred eighty-eight patients were included. The average age at ALL diagnosis was 7.7±4.8 (0.3–17.8) years, with 114/188 (60.6%) male patients. Among the cohort, 1.6% had Down syndrome, 73.9% exhibited B-cell lineage, and 5.3% were Ph+. Host and disease characteristics are summarized in Tables 1 and 2.

**Table 1:** Host and disease characteristics of 188 acute lymphoblastic leukemia patients according to the occurrence of hyperglycemia.

		Hyperglycemia		p-value
		No (n=106)	Yes (n=82)	
Chronologic age at diagnosis (years)				0.724^b^
	Mean±SD	7.6±4.5	7.9±5.2	
	Median (min–max)	7.3 (0.3–17.8)	6.3 (1.1–17.9)	
Gender, n (%)				0.085
	Female	36 (48.6)	38 (51.4)	
	Male	70 (61.4)	44 (38.6)	
Ethnicity, n (%)				0.451
	Caucasian	55 (59.1)	38 (40.9)	
	Non-Caucasian	51 (53.7)	44 (46.3)	
Puberty at diagnosis, n (%)				0.120
	No	73 (58.9)	51 (41.1)	
	Yes	10 (41.7)	14 (58.3)	
	N/A	23	17	
Nutritional status at diagnosis, n (%)				0.874^a^
	Underweight	4 (50.0)	4 (50.0)	
	Healthy	73 (55.3)	59 (44.7)	
	Overweight	16 (64.0)	9 (36.0)	
	Obesity	13 (56.5)	10 (43.5)	
Family history of diabetes, n (%)				0.828
	No	70 (56.0)	55 (44.0)	
	Yes	26 (54.2)	22 (45.8)	
	N/A	10	5	
Cellular lineage, n (%)				0.959^a^
	B-cell	78 (56.1)	61 (43.9)	
	T-cell	25 (58.1)	18 (41.9)	
	Mixed	3 (50.0)	3 (50.0)	
Chromosome Ph+, n (%)				0.336^a^
	No	102 (57.3)	76 (42.7)	
	Yes	4 (40.0)	6 (60.0)	
Risk of relapse, n (%)				0.064
	Low	6 (42.9)	8 (57.1)	
	Intermediate	30 (71.4)	12 (28.6)	
	High	70 (53.0)	62 (47.0)	
Protocol, n (%)				0.962
	GBTLI-2009	65 (56.5)	50 (43.5)	
	BFM	41 (56.2)	32 (43.8)	

Ph: Philadelphia; GBTLI: Brazilian Group for the Treatment of Leukemia in Childhood; BFM: Berlin-Frankfurt-Munich protocol. SD: standard deviation; N/A: not available. p-value–chi-square descriptive level,

a Fisher's Exact test,

b Student's t-test.

**Table 2:** Host and disease characteristics of acute lymphoblastic leukemia patients according to treatment protocol.

		GBTLI-2009 (n=115; 61.2%)	BFM (n=73; 38.8%)	Total (n=188)	p-value
Chronologic age at diagnosis (years)					0.170^b^
	Mean±SD	7.3±4.7	8.3±4.8	7.7±4.8	
	Median (min–max)	6.5 (0.3–17.9)	7.4 (1.1–17.8)	6.9 (0.3–17.9)	
Gender, n (%)					0.488
	Female	43 (37.4)	31 (42.5)	74 (39.4)	
	Male	72 (62.6)	42 (57.5)	114 (60.6)	
Ethnicity, n (%)					0.067
	Caucasian	63 (54.8)	30 (41.1)	93 (49.5)	
	Non-Caucasian	52 (45.2)	43 (58.9)	95 (50.5)	
Puberty at diagnosis, n (%)					0.236
	No	78 (86.7)	46 (79.3)	124 (83.8)	
	Yes	12 (13.3)	12 (20.7)	24 (16.2)	
	N/A	25	15	40	
Nutritional status at diagnosis, n (%)					0.159^a^
	Underweight	6 (5.2)	2 (2.7)	8 (4.3)	
	Healthy	74 (64.3)	58 (79.5)	132 (70.2)	
	Overweight	17 (14.8)	8 (11.0)	25 (13.3)	
	Obesity	18 (15.7)	5 (6.8)	23 (12.2)	
Family history of diabetes, n (%)					0.137
	No	76 (68.5)	49 (79.0)	125 (72.3)	
	Yes	35 (31.5)	13 (21.0)	48 (27.7)	
	N/A	4	11	15	
Cellular lineage, n (%)					0.073^a^
	B-cell	91 (79.1)	48 (65.8)	139 (73.9)	
	T-cell	20 (17.4)	23 (31.5)	43 (22.9)	
	Mixed	4 (3.5)	2 (2.7)	6 (3.2)	
Chromosome Ph+, n (%)					0.514^a^
	No	110 (95.7)	68 (93.2)	178 (94.7)	
	Yes	5 (4.3)	5 (6.8)	10 (5.3)	
Risk of relapse, n (%)					0.959
	Low	9 (7.8)	5 (6.8)	14 (7.5)	
	Intermediate	26 (22.6)	16 (21.9)	42 (22.3)	
	High	80 (69.6)	52 (71.3)	132 (70.2)	
Hyperglycemia, n (%)					0.962
	No	65 (56.5)	41 (56.2)	106 (56.4)	
	Yes	50 (43.5)	32 (43.8)	82 (43.6)	

GBTLI: Brazilian Group for the Treatment of Leukemia in Childhood; BFM: Berlin-Frankfurt-Munich protocol; SD: standard deviation; N/A: not available; Ph: Philadelphia. p-value–chi-square descriptive level,

a Fisher's Exact test,

b Student's t-test.

### Hyperglycemia

Hyperglycemia was observed in 82/188 (43.6%) patients, with 90.2% of episodes occurring during the induction phase. No significant differences were found between the euglycemic and hyperglycemic groups regarding host and disease characteristics (p>0.050). All patients with hyperglycemia received *E. coli* native ASP. Furthermore, no differences were found in the cumulative doses of ASP (42,648.6±33,537.1 vs. 75,625.0±69,279.0; p=0.171 [IU/m^2^]) or PRED (730.2±363.9 vs. 863.5±561.1; p=0.408 [mg/m^2^]) between GBTLI-2009 and BFM until hyperglycemia onset. However, GBTLI-2009 employed lower doses of DEX than BFM (52.5±43.9 vs. 256.7±155.1; p=0.023 [mg/m^2^]).

Transient subcutaneous insulin was required for 15.8% of patients with hyperglycemia. One male subject required permanent subcutaneous insulin following pancreatitis during ALL therapy despite lacking type 1 DM autoimmune markers (anti-GAD and IA-2).

### Hyperglycemia predisposing factors

The following variables were identified as risk factors for hyperglycemia, and are detailed as odds ratio (OR), and 95% confidence interval (95%CI): age at ALL diagnosis (OR 0.86; 95%CI 0.75–0.99; p=0.039), puberty (OR 7.94; 95%CI 1.45–43.64; p=0.017), and risk of relapse (OR 0.33; 95%CI 0.14–0.78; p=0.011). The risk of hyperglycemia in pubertal patients was 7.9 times greater than in prepubertal counterparts. Additionally, patients classified as intermediate risk for relapse exhibited a diminished risk of hyperglycemia compared to high-risk patients. Refer to Table 3.

**Table 3:** Logistic regressions for hyperglycemia risk factors among acute lymphoblastic leukemia patients during chemotherapy.

		Univariate model		Initial multivariate model		Final multivariate model	
		Odds ratio Brute (95%CI)	*p-value*	Odds ratio Adjusted (95%CI)	*p-value*	Odds ratio Adjusted (95%CI)	*p-value*
Age at ALL diagnosis (years)		1.01 (0.95–1.07)	*0.718*	0.85 (0.72–1.01)	*0.066*	0.86 (0.75–0.99)	***0.039***
Female (ref.=Male)		1.68 (0.93–3.03)	*0.086*	1.64 (0.71–3.77)	*0.244*	–	–
Non-Caucasian (ref.=Caucasian)		1.25 (0.70–2.22)	*0.451*	1.69 (0.79–3.64)	*0.180*	–	–
Puberty		2.00 (0.83–4.86)	*0.124*	10.13 (1.34–76.40)	***0.025***	7.94 (1.45–43.64)	***0.017***
Nutritional status (ref.=Healthy)			*0.854*		*0.550*		
	Underweight	1.24 (0.30–5.16)	*0.770*	0.41 (0.06–2.80)	*0.366*	–	–
	Overweight	0.70 (0.29–1.69)	*0.423*	0.48 (0.14–1.68)	*0.253*	–	–
	Obesity	0.95 (0.39–2.33)	*0.914*	1.13 (0.35–3.62)	*0.839*	–	–
Family history of DM		1.08 (0.55–2.10)	*0.828*	1.27 (0.54–2.98)	*0.576*	–	–
Cellular lineage (ref.=B-cell)			*0.925*		*0.627*		
	T-cell	0.92 (0.46–1.84)	*0.815*	0.72 (0.26–2.02)	*0.539*	–	–
	Mixed	1.28 (0.25–6.56)	*0.768*	0.38 (0.04–3.52)	*0.392*	–	–
Chromosome Ph+		2.01 (0.55–7.38)	*0.291*	0.78 (0.04–16.29)	*0.875*	–	–
Risk of relapse (ref.=High)			*0.069*		*0.073*		***0.020***
	Low	1.51 (0.49–4.58)	*0.471*	1.11 (0.25–4.84)	*0.893*	1.46 (0.43–4.94)	*0.539*
	Intermediate	0.45 (0.21–0.96)	***0.038***	0.35 (0.14–0.90)	***0.029***	0.33 (0.14–0.78)	***0.011***
BFM protocol (ref.=GBTLI-2009)		1.02 (0.56–1.83)	*0.962*	0.93 (0.42–2.08)	*0.861*	–	–

ALL: acute lymphoblastic leukemia, 95%CI: confidence interval of 95%, DM: diabetes mellitus, Ph: Philadelphia, BFM: Berlin-Frankfurt-Munich, GBTLI: Brazilian Group for the Treatment of Leukemia in Childhood. n=138 and n=148, respectively for initial and final multivariate models. Hosmer and Lemeshow tests–Initial multivariate model (p=0.292) and final (p=0.839). p-values are indicated as italic values. Bold values are statistically significant.

### Infection

One hundred fourteen out of 188 (60.6%) patients experienced infection episodes during ALL treatment. Nonetheless, hyperglycemia did not emerge as a risk factor for infection during ALL treatment (OR 0.62; 95%CI 0.31–1.26; p=0.189).

### Relapse

By the conclusion of data collection, 57/188 (30.3%) patients had experienced a relapse of ALL, with no statistically significant difference between the euglycemic and hyperglycemic cohorts [data are reported as hazard ratio (HR) and 95%CI] (HR 1.32; 95%CI 0.78–2.23; p=0.302). Notably, despite hyperglycemia, females exhibited a 73% reduced risk of ALL relapse compared to their male counterparts (HR 0.27; 95%CI 0.11–0.66; p=0.004).

### Mortality

By the end of data collection, 58/188 (30.8%) patients succumbed to ALL or its secondary complications, and mortality occurred prematurely. The incidence of mortality did not differ significantly between the euglycemic and hyperglycemic groups (HR 1.44; 95%CI 0.84–2.45; p=0.185). However, the risk of mortality in patients treated with the BFM protocol was 2.3 times higher compared to GBTLI (HR 2.26; 95%CI 1.25–4.06; p=0.007). In addition, patients with overweight or obesity faced a mortality risk of 3.2 (HR 3.18; 95%CI 1.47–6.91; p=0.003) and 2.4 (HR 2.41; 95%CI 1.05–5.51; p=0.037) times greater than those of average weight, respectively. Additionally, patients with up to two episodes of infection and those exhibiting elevated pancreatic enzyme levels had a mortality risk of 2.5 (HR 2.47; 95%CI 1.25–4.87; p=0.009) and 2.8 (HR 2.81; 95%CI 1.35–5.84; p=0.006) times more pronounced. The overall incidence of acute pancreatitis was 22/188 (11.7%) and occurred primarily during the induction phase.

## DISCUSSION

This investigation scrutinized the risk factors associated with hyperglycemia during chemotherapy for childhood ALL. Key determinants influencing hyperglycemia included age at diagnosis, the onset of puberty, and an intermediate risk of relapse. Nevertheless, hyperglycemia did not correlate with disease relapse, infections, or mortality.

The incidence of hyperglycemia during ALL treatment was recorded at 43.6%, surpassing previous studies involving pediatric populations, which reported rates ranging from 3.8 to 20%^2–4,18^. However, one study recorded a hyperglycemia incidence of 58%^5^, likely due to the inclusion of random and postprandial blood glucose levels in their analysis.

The glucose threshold for defining hyperglycemia varied across studies. This study's cutoff was lower than others (>7.0 vs. 7.8 or 11.1 mmol/L). It is also crucial to note that most existing literature comprises retrospective studies, which may not have accounted for mild and asymptomatic glycemic fluctuations^2–5,19^.

Most hyperglycemic episodes transpired during the induction phase (90.2%), irrespective of the treatment protocol employed. This phenomenon may be attributed to the synergistic effects of glucocorticoids and ASP, as reported in various studies^2,4–6,18–20^.

The role of glucocorticoid type in influencing hyperglycemia remains contentious. Across different protocols, the incidence of hyperglycemia did not vary significantly despite a higher cumulative dose of DEX in patients treated under the BFM protocol^2,4,19^. This observation suggests that individual intrinsic characteristics are pivotal in the incidence of hyperglycemia, as glucose fluctuations may represent a synergistic interplay of personal traits, disease state, and therapeutic interventions^2,3,5,6^.

The comparison of various ASP formulations and their impact on hyperglycemia was not conducted, as all patients received *E. coli* native ASP. Nonetheless, prior studies indicated that *E. coli* native ASP was linked to a heightened risk of hyperglycemia compared to PEG ASP^4,21^.

In evaluating hyperglycemia risk factors, age at diagnosis and the onset of puberty were significant, likely due to insulin resistance^2,6^. Although patients over 10 years of age are deemed at risk for hyperglycemia, the predominance of individuals not yet in puberty in this cohort (83.8%) may have acted as a confounding variable. Furthermore, other studies have assessed patients solely by chronological age without considering pubertal status^4–6,18,22,23^.

Patients classified as having an intermediate risk for relapse exhibited a lower likelihood of hyperglycemia compared to those at high risk of recurrence, aligning with findings from other studies^22,23^. No differences in hyperglycemia occurrence have been noted when contrasting low- and high-relapse-risk patients, likely due to the limited number of individuals classified as low-risk (7.4%) in our study population. Conversely, other studies did not feature relapse risk stratification as a potential contributor to hyperglycemia^5,20^.

Previous literature has identified additional clinical variables as risk factors for transient hyperglycemia, including female gender, patients with overweight and obesity, family history of DM, and Down syndrome^2,4-6,22^. However, in this study, these variables did not retain statistical significance.

Despite hyperglycemia, only 15.9% of individuals demanded transient insulin therapy, corroborating findings from other studies. Notably, one patient developed pancreatitis concomitant with hyperglycemia, demanding permanent insulin therapy. Nonetheless, pancreatitis is an independent event and does not explain all the episodes of hyperglycemia so far^5^.

According to McCormick et al.^22^, the need for insulin therapy is heightened in patients with pancreatitis, as compromised pancreatic β-cell function may contribute to persistent insulin deficiency. The potential toxic effects of elevated glucose levels, even transiently, on pancreatic cells remain uncertain^23^.

Research involving adults with hyperglycemia during ALL treatment suggests poorer outcomes; however, results in the pediatric population remain inconclusive. Hyperglycemia as an adverse effect did not correlate with increased severe infections in this population^8,10^.

No differences in relapse rates were observed between euglycemic, mild, or severe hyperglycemia groups. However, the severity of hyperglycemia may influence the risk of ALL recurrence, contingent upon the glucose cutoff measurement utilized for this analysis^5,23^.

Mortality rates did not differ between euglycemic and hyperglycemic groups. Nonetheless, this remains a contentious issue, as several studies have indicated that the probability of survival five years post-ALL diagnosis is more significant among euglycemic individuals^5,23,24^.

A significant limitation of this study stems from its retrospective nature, potential confounders, limited generalizability due to the single institution setting, and the lack of available data regarding glycemic decompensation, including pancreatic function and details of acute complications (such as diabetic ketoacidosis and hyperosmolar hyperglycemic state). Even though there was no control group, hyperglycemic patients were compared to euglycemic individuals.

## CONCLUSION

In summary, hyperglycemia constitutes an early adverse event in the treatment of ALL, with a notable incidence among patients undergoing chemotherapy. Rigorous glycemic monitoring, particularly during the induction phase of ALL treatment, may facilitate the early identification of glycemic metabolism impairments, thereby enabling improved management of these patients and mitigating risks during follow-up.

## Data Availability

The datasets generated and/or analyzed during the current study are available from the corresponding author upon reasonable request.
